# Automatic detection and resolution of measurement-unit conflicts in aggregated data

**DOI:** 10.1186/1755-8794-7-S1-S12

**Published:** 2014-05-08

**Authors:** Soroush Samadian, Bruce McManus, Mark Wilkinson

**Affiliations:** 1 UBC James Hogg Research Center, Institute for Heart + Lung Health, Room 166 - 1081 Burrard Street, St. Paul's Hospital Vancouver, BC, Canada, V6Z 1Y6; 2 Centro de Biotecnología y Genómica de Plantas, Universidad Politécnica de Madrid, Madrid, España

## Abstract

**Background:**

Measurement-unit conflicts are a perennial problem in integrative research domains such as clinical meta-analysis. As multi-national collaborations grow, as new measurement instruments appear, and as Linked Open Data infrastructures become increasingly pervasive, the number of such conflicts will similarly increase.

**Methods:**

We propose a generic approach to the problem of (a) encoding measurement units in datasets in a machine-readable manner, (b) detecting when a dataset contained mixtures of measurement units, and (c) automatically converting any conflicting units into a desired unit, as defined for a given study.

**Results:**

We utilized existing ontologies and standards for scientific data representation, measurement unit definition, and data manipulation to build a simple and flexible Semantic Web Service-based approach to measurement-unit harmonization. A cardiovascular patient cohort in which clinical measurements were recorded in a number of different units (e.g., mmHg and cmHg for blood pressure) was automatically classified into a number of clinical phenotypes, semantically defined using different measurement units.

**Conclusions:**

We demonstrate that through a combination of semantic standards and frameworks, unit integration problems can be automatically detected and resolved.

## Background

Integration, comparison and interpretation of quantitative data require, as a first step, that all measurements are represented in the same units. Discordance in units is common in integrative research, is difficult to detect, and has severe consequences when not managed effectively. Even NASA has made serious and expensive errors by failing to detect and account for measurement-unit conflicts[1].

This problem is well-recognized in clinical research, due to its complex, multi-dimensional and heterogeneous nature, and where highly disparate datasets, often from non-coordinating groups, need to be brought together. This work, therefore, is contextualized within a clinically-oriented study in which we would be required to gather clinical data from a number of participating groups, and attempt to automatically categorize individual patients over existing health-risk guidelines using semantic technologies[2]. Prior to undertaking the study, we became aware of the potential for measurement unit conflicts in these integrated datasets. Rather than creating an *ad hoc *solution, we attempted to define a lightweight, standards-compliant, and semantics-based solution that could be re-used by other bio/medical research projects.

It should be noted that, in the current work, it was not our intention to propose novel epistemic theories of qualities and measurements as it is both beyond the scope of our present work, and not necessarily required to achieve our major objectives in the current study which was to focus on providing practical and operational solution for clinicians and other health researchers using existing standards and theoretical frameworks. The reader more interested in detailed core theoretical foundations on which this study (and many existing ontologies such as DOLCE) is based, is referred to (among others) [3-5].

The rest of this paper is organized as follows. We will explain the challenges associated with formal measurement-unit representation and integration in the Semantic Web, and discuss related tools and resources. We describe possible design-choices for modeling units, and the problems or benefits of these alternatives. We then discuss our proposed framework, and justify the core design and representation choices through presentation of a case study of patient phenotype classification within the cardiovascular domain. Finally, concluding remarks together with future extensions will be presented.

## Related work

Various standards exist for unit representation, the most notable of which (in the Western world) is the International System of Standards (SI), now adopted in all areas of science[6]. Nevertheless, the choice of units, even within this system, is sufficiently broad that reliable automated integration of quantitative measurements remains problematic [7], and as a result, a consistent methodology to interpret and integrate the units within and between datasets remains to be established.

Recently, semantic solutions in the form of measurement unit ontologies have emerged as a potential path towards a solution. These ontologies generally use the Resource Description Framework (RDF) [8] for data encoding and Web Ontology Language(OWL)[9] to encode axioms that can be used for automated inference over the data. However, while defining standards for the representation of numerical/quantitative data, the RDF standard does not inherently define an approach to representing the measurement units associated with that data [10]. Additionally, these ontologies are often domain-specific, and therefore have limited coverage of the full range of measurement units - focusing only on units relevant to that domain of investigation - and lack informative relationships between related units. For instance, the GALEN concept *MilligramPerDeciLiter*, is defined as a subclass of the concepts *ConcentrationUnit*, however it lacks any indication that this unit is composed of combination of two base units (gram and liter) and two prefixes (milli and deci).

Recently, ontologies have been developed to more generically address formalizations of unit representation and integration. Prominent examples of such ontologies are the Measurement Unit Ontology (MUO)[10], Ontology for Engineering Mathematics(EngMath)[11], Quantities, Units, Dimensions and Types (QUDT)[12], and the Ontology of Units of Measure (OM) [13]. Their salient features are (for a more comprehensive review please refer to the [14]):

***MUO***: In MUO, complex measurement units can be derived from the base ones in a modular fashion. MUO proposes a convenient framework for defining new units of measurements in terms of existing ones. However, while MUO defines metric prefixes (e.g., centi- and kilo-), which could be used to automate automated conversion for SI-based measurements, it lacks quantitative or formula-based definitions for converting between SI units and similar qualities in other unit-systems (e.g., inch and cm).

***Ontology for Engineering Mathematics(EngMath): ***EngMath is an ontology for mathematical modelling in engineering, written in Ontolingua[15]. It provides conceptual foundations for representing mathematical and physical entities such as scalars, vectors, tensors, physical quantities, physical dimensions and units explicitly designed for knowledge sharing applications in engineering[11].

Regarding the unit representation problem, the main feature in EngMath (absent from MUO) is the component "physical dimensions". The physical dimension of a quantity is an abstraction of a quantity ignoring magnitude, sign and direction aspects[13]. The dimension of a quantity can be thought of as independent set of base dimensions[13]. For instance the quantity Body Mass Index (BMI) has the dimension that can be decomposed into base dimensions *mass *and *length:*$ML^{-2}$. The base dimensions in SI systems are length (L), mass (M), time (T), electric current (I), temperature (04E8), amount of substance (N) and luminous intensity (J).

EngMath (as opposed to MUO and UO) provides the enough semantic information to convert many of unit-pairs of the same dimension that are either defined as basic units or composed from the basic units[16]. The key limitation of EngMath for our purposes is that it is not available in OWL. This problem is addressed in two more recent ontologies QUDT and OM that use a similar conceptual framework.

***QUDT***: QUDT defines "quantity dimensions" which allows for automatic consistency checking of different quantities. QUDT also includes several major unit-systems such as the CSG system of Units, SI and others[17], but relates all other unit systems back to SI using two data properties - "*conversion offset*", "*conversion multiplier*" - that could enable automated conversion between any non-SI-based unit and its SI-based equivalent. In terms of coverage for base units QUDT is fairly comprehensive; however it lacks a number of derived units (e.g., the centimeters of mercury column commonly used for clinical measurements of blood pressure); however it provides the framework within which these units could be created.

**Figure 1:**
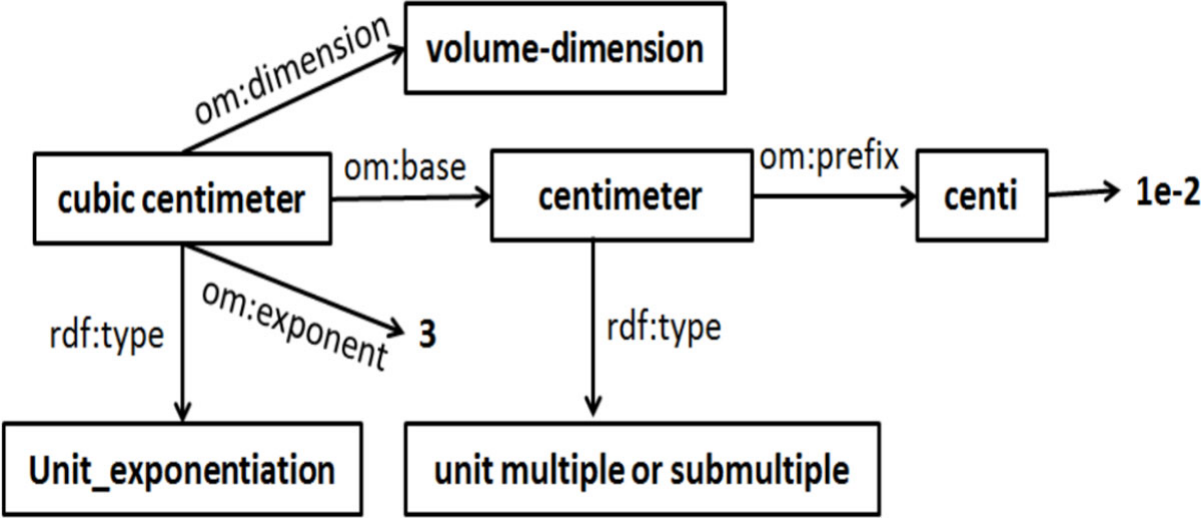
**OM representation of cubic centimeter**. The "compound units" in OM are divided into three top categories: "unit division", "unit multiplication" and "unit exponentiation" which provides additional information for automatically calculating the dimensions of new units.

***OM: ***OM and QUDT are similar in terms of high level design features and hence we only discuss the key differences. One notable difference, is that OM defines "Quantity kinds" (e.g., acceleration and absorption) as OWL classes, which facilitates logical reasoning[13]. Another advantage of OM -over QUDT- is that it uses the SI prefixes to relate submultiples (e.g., *deci*) and multiples (e.g., *deca*) of units. Additionally, OM defines the relationship between compound units (e.g., kilogram per cubic meter) and their individual constituents (kilogram and cubic meter). Moreover, "compound units" is further divided into "unit division" (e.g., meter per second), "unit exponentiation" (e.g., meter squared) and "unit multiplication" (e.g., meter kilogram). For instance, for the unit "millimole per cubic centimeter" (mmol/cm3), the nominator and denominator are defined as "millimole" and "cubic centimeter", respectively, where millimole is related to mole by Prefix milli (om:factor = 1e-3) while "cubic centimeter" is an instance of "unit exponentiation". This additional feature in OM allows for the automated calculations of dimensions for new units in based on their constituents. Figure 1 shows the model for "cubic centimeter", revealing the relationship between OM classes that enable automated reasoning and unit inter-conversion.

In addition to above advantages, OM provides Web Services that can be used programmatically to incorporate knowledge in OM in other applications making it suitable for our study.

## Methods

### Dataset and data collection

The dataset used for these investigations included the clinical records of a cardiovascular patient cohort collected from a referral hospital in Nebraska, USA, between 1986 and 1989, including 536 unique patients. Table 1 shows some columns from two rows of the dataset used in this study. The intended meaning of acronyms for each column header (e.g., SBP for *Systolic Blood Pressure*) was confirmed with the clinician who had originally collected the dataset. In the last four columns "1" represents "high risk" and "0" represents a "low risk" for the condition listed in the header.

**Table 1:** Snapshot of the Dataset.

ID	HEIGHT	WEIGHT	SBP	CHOL	HDL	BMI GR	SBP GR	CHOL GR	HDL GR
**pt1**	***1.82***	**177**	***128***	***227***	***55***	**0**	**0**	**1**	**0**
**pt2**	***179***	**196**	***13.4***	***5.9***	***1.7***	**1**	**0**	**1**	**0**

The first two rows of the dataset used in the original format. In the last four columns "1" represents "high risk" and "0" represents a "low risk" for the condition listed in the header

As shown in the table, this dataset exhibited several features that presented significant challenges to automated integration and annotation and that are common with legacy (and even contemporary) clinical data, including:

1) The measurement-units were not represented explicitly.

2) Different rows of the same data set were represented in different measurement units shown in different colors. For instance, HDL is represented in *milligram/deciliter *(*Italic font*) in the first row and in *mmol/liter *(*Italic font*) in the second row shown in different colors)

3) The system of units used, even in the same row, could change (e.g., height in SI and weight in Imperial units in the first row).

These observations highlight the need for a practical solution to ameliorate the problem of measurement-unit conflict resolution in health care.

### Data transformation

#### Ontologies and standards used

***OM: ***As stated, OM provides a rich conceptualization of the compound units common in clinical data, we selected OM as our preferred unit-ontology starting point.

***GALEN: ***GALEN [18] is a rich compositional ontology of the medical domain, covering anatomy, function, diseases, symptoms, drugs, and procedures. Following an approach published previously[2] we re-factoring and extending a number of cardiovascular-relevant classes of GALEN such that they could be used for logical reasoning and classification.

***SADI and SHARE: ***SADI[19] is a set of standards-compliant design principles for exposing stateless Web Services on the Semantic Web. SADI Services consume and produce RDF data, where the input and output data properties are described by OWL classes. These classes are, similarly, utilized to discover Services of interest through their registration in the SADI Service registry. SHARE[19] is an enhanced SPARQL query engine which is capable of (a) decomposing OWL classes into their constituent property restrictions, and (b) discovering and invoking SADI Services based on the properties those Services consume/produce.

***SIO: ***The SemanticScience Integrated Ontology (SIO) is an ontology that provides models for the representation of the scientific data [20]. It includes design principles that facilitate creation of flexible software and is extensively used by analytical tools exposed using SADI Semantic Web Services. Therefore our adoption of SIO in this work allows us to more easily take advantage of existing resources published using the SADI design pattern, as well as rapidly publish and integrate new tools as-needed.

#### Clinical data modeling

The GALEN ontology does not define the structure and properties of individuals who would be members of its ontological classes, and therefore cannot easily be used to classify data. As such, we extended the concepts in GALEN using SIO and OM. For example, we extend the concept SystolicBloodPressure in GALEN as in OWL as follows:

measure:SystolicBloodPressure =

        **galen**:SystolicBloodPressure *and*

              ("**sio**:has measurement value" *some*

                  "**sio**:measurement" *and*

                        ("**sio**:has unit" *some *"**om**:unit of measure") *and*

                        (***"*om**:dimension" *value *"**om**:pressure dimension") *and*

                        "**sio**:has value" *some *rdfs:Literal))

**Figure 2:**
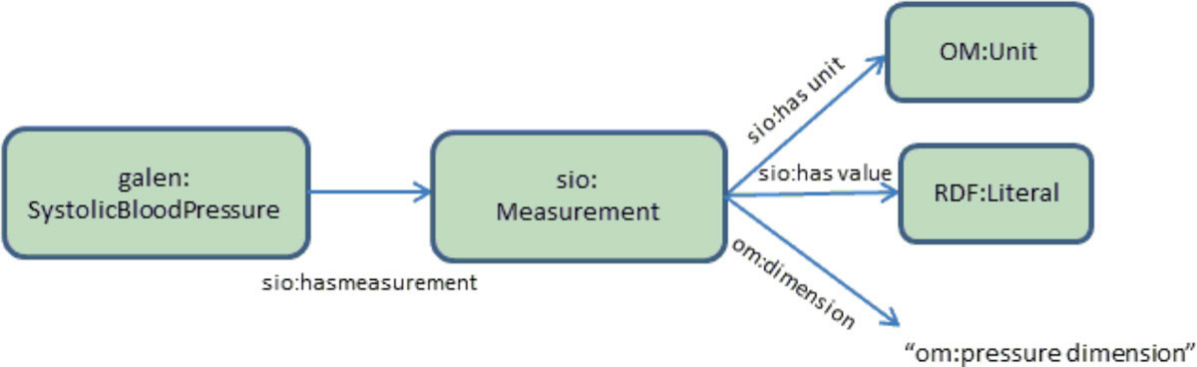
**Extending clinical concepts**. Extending clinical concepts in GALEN with richer logic including measurement values and units. Once extended, Galen classes can be used for semantically enriched analyses.

In the above, the measurement units are linked to OM's "pressure dimension". This guarantees dimension compatibility during logical reasoning; i.e., all pressure data in the clinical dataset are associated to "pressure dimension" by *om:dimension *relationship, and can therefore automatically be directed to pressure-dimension-relevant conversion services. Figure 2 shows the schematic view of the data model for systolic blood pressure. The model provides a machine-processable mechanism for expressing semantics of measurement-units in the clinical domain.

We followed OM's framework for defining new units in order to create several unit-types commonly used in clinical science, but missing from OM's existing set. For instance the unit "*centimeter_of_mercury_column*" (*cmHg*) which is often used for monitoring blood pressure does not exist in OM and was defined according to OM's standards.

#### Semantic Web services

A single generic SADI-compliant Web Service was constructed to manage conversions over the major quantities (dimensions) of measurement most frequently used in clinical setting including *Pressure, Concentration, Temperature, Length*, and *Mass*. We first discuss the generic signature of the service and then describe an example use cases for pressure and concentration (with minor differences) in more details. The other dimensions follow similar principles.

The SADI service uses the semantics of the input data it receives to automatically configure itself to the correct type of conversion. The input and output of this service are as follows:

Input:

        "sio:has measurement value" ***some***

              "sio:has unit" ***some ***"om: Unit of measure" ***and ***"sio:has value" ***some ***rdfs:Literal

Output:

          "sio:has measurement value" ***some***

                "sio:has unit" ***some ***"om: Unit of measure" ***and ***"sio:has value" ***some ***rdfs:Literal

                    ***and ***"om:has dimension" ***exactly 1 ***om:Dimension

The following algorithmic steps are carried out automatically for each input data that meet the requirement:

1. Find the dimension of input data. If input data is not explicitly annotated with dimension, use the web service provided by OM to annotate the dimension of the input data unit.

2. Use the dimension information to automatically configure the service to the appropriate conversion (e.g., *Pressure *convertor service).

3. Use the dimension information as the input to find all the compatible units (units with the same dimension)

4. Iterate through compatible units frequently used in clinical settings (e.g., iterate through all units for *Pressure*) and apply the conversion for each (the limitations of this approach are discussed below)

5. Use the API to calculate "*unit conversion offset"*(getConversionOffset) and "*unit conversion factor"*(getConversionFactor) to convert the input data into selected compatible units. Attach the dimension and unit/value pairs to the output.

## Results

### Pressure

All patient data was converted into RDF format using a pattern similar to that shown in Figure 2. We then defined an ontological class "High-Systolic-Blood-Pressure-Measurement"[21], as follows:

        measure:SystolicBloodPressure ***and***

              sio:hasMeasurement ***some***

                   (sio:Measurement ***and ***("sio:has unit" ***value ***om:kilopascal) ***and***

                      (sio:hasValue some double[>= "18.7"^^double])))

This model uses units (kilopascals) that differ from those in our dataset (mmHg and cmHg), which allows us to demonstrate the ability of the system to automatically detect and resolve unit conflicts. A SPARQL query is provided to the SHARE query client that searches for high blood pressure measurements, as follows:

    SELECT ?record ?convertedvalue ?riskgrade

    FROM <./patient.rdf> WHERE

        {

            ?record rdf:type measure:**HighSystolicBloodPressure**.

            ?record sio:hasMeasurement ?measurement.

            ?measurement sio:hasValue ?convertedvalue.

            ?record cardio:ExpertClassification ?riskgrade.

        }

In the above query SHARE examines the HighSystolicBloodPressure class and discovers the "sio:has unit" ***value ***om:kilopascal axiom, indicating that measurements of High Systolic Blood Pressure will need to be expressed in kilopascals. It compares this to the measurements in its dataset, and logically determines that they are discrepant in their units. It then queries the SADI registry to find a Service (the single Service described above) that provides conversions on the OM dimension of "Pressure" - i.e., a unit-conversion service with the input property of some "om:pressure dimension". It passes the measurement data to that service for unit-homogenization. For each individual incoming measurement, the unit is examined and the offset and coefficient parameters required to convert between source (input data) and target (required by the OWL class) unit are dynamically retrieved by the service using the OM REST interface. The conversion calculation is then synthesized and applied to the incoming data. Once all data have been processed, the result is a data-set with all pressure units harmonized as kilopascals. The resulting output data is integrated into the local knowledge-base, prior to undertaking logical reasoning using the Pellet reasoned, that classifies the patient data as being consistent or inconsistent with the HighSystolicBloodPressure ontological definition.

Table 2 shows a demonstrative sub-set of the result data corresponding to HighSystolicBloodPressure individuals, the showing a variety of pressure units being homogenized the kilopascal in accordance with the OWL class definition. Moreover, the system was able to identify all 134 individuals, classified by the expert as having high systolic blood pressure, with no false positives. It should be noted that though we did not find any misclassification, the possibility of rounding errors introduced as a result of conversion cannot be ruled out; thus in future iterations it may be desirable to specifically engineer the conversion services to make "sensible" choices about rounding, based on advice from expert clinicians.

**Table 2:** Units and values.

RecordID		Start Val		Start Unit		End Val		End Unit	Expert's classification
cm_hg1		15		cmHg		19.998		**kilopascal**	**High**
cm_hg2		14.6		cmHg		19.465		**kilopascal**	**High**
mm_hg1		148		mmHg		19.731		**kilopascal**	**High**
mm_hg2		146		mmHg		19.465		**kilopascal**	**High**

Units and valuse before and after conversion (2 digit precision).

### Concentration

The term concentration most frequently refers to amount of substance in a solution. There are two major types of units that are used to represent concentration in clinical settings usually used to denote the concentration of different chemicals in plasma (e.g., Hemoglobin). The first type represents the amount of substance per unit volume (e.g., gram per liter) and the second type represents the number of moles per unit volume (e.g., mole per liter). Thus, we should note that, for concentration, both dimensions of *mole/m3 *($NL^{-3}$) and *kg/m3 *($ML^{-3}$) are used in practice. Molar-based units are routinely used in medicine and physiology. As a result 1) the "conversion factor" between these different dimensions is not a dimensionless parameter and 2) the conversion factor between depends on the molar mass of the specific molecule for which the measurement has been made. For example to convert *mmol/L *to *mg/dL *for Triglyceride and HDL we need to multiply by 88.57 and 38.67, respectively. A comprehensive list of molar-based units for plasma concentration of different chemicals and their corresponding conversion factor can be found in [22].

To achieve conversions in this case, it is necessary to know the specific type of incoming measurement, based on its GALEN class; for example, if it is a Triglyceride measurement, the incoming measurement must be of rdf:type galen:tryglyceride, since the incoming units carry insufficient semantics in-and-of themselves to generate the calculation parameters. Once the relevant compound is determined from the logical type of the incoming measurement, the conversion constants are calculated using the molecular mass of each molecule as per the look-up table in [22]. Here we only implemented the concentration molecule-types used most frequently in clinical sciences (see supplementary materials) for performance reasons; however the framework is extensible by expanding the look-up table and the galen molecule type ontology.

Evaluations similar to those described for Systolic Blood Pressure were carried-out for Cholesterol and HDL classification, and the system was able to correctly convert and classify all records.

### Complex Risk Classification (revisited)

As mentioned, in our previous study[2], we demonstrated that the combination of semantically-explicit data, logically rigorous models of clinical guidelines, and publicly-accessible Semantic Web Services, can be used to execute automated, rigorous and reproducible clinical classifications. However, in the previous study our unit conversion services were written separately for each quantity (one service for Pressure one service for Concentration and so on). In addition, the conversion formulas for conversion between different units were hard-coded within the Web Services. Additionally, using our previous framework the relation between a quantity and its dimension was coded manually. In the current experiment, we addressed these shortcomings and repeated the pervious experiment. Not surprisingly, the results for binary classifications were consistent with previous experiment (see supplementary materials). In the following we will work through classification of patients based on their BMI value. The other risk classifications follow a similar pattern and are presented in the supplementary materials.

Modeling BMI risks is more complex than modelling concentration, since BMI is derived by algorithmic analysis of other core measurements (Height and Weight). BMI is calculated using a person's weight and height. BMI in SI is calculated using the following formula.

$$BMI=\frac{mass\left(kg\right)}{{\left(height\left(m\right)\right)}^{2}}=\frac{mass\left(lb\right)}{{\left(height\left(in\right)\right)}^{2}}\times 703$$

Once again, for evaluation purposes we intentionally defined BMI in a different unit than the one presented in the dataset. Due to reasons not discussed here (see [2]), the clinical researcher had used a different cut-off threshold than the ones suggested by American Heart Association(AHA) (26 kg/m2 was used instead of 25 kg/m2 to classify patient as "Overweight").

    Overweight =

        Patient ***and***

            (sio:hasAttribute ***some***

                (measure:BodyMassIndex ***and***

                    sio:hasMeasurement ***some ***( sio:Measurement and

                    (sio:hasUnit ***value ***om:pound-per-inch- squared) ***and***

                        (sio:hasValue ***some ***double[>= 18278.0]))))

where as before measure:BMI is extended using BMI class in galen ontology and 18278 (the production of 26 and 703) cut-off threshold is used to stratify patients. Subsequently, a SPARQL query was composed to classify records based on their BMI value.

    SELECT ?record ?convertedvalue ?riskgrade

    FROM <./patient.rdf> WHERE

        {

            ?record rdf:type measure:**Overweight**.

            ?record sio:hasMeasurement ?measurement.

            ?measurement sio:hasValue ?convertedvalue.

            ?record cardio:ExpertClassification ?riskgrade.

        }

**Figure 3:**
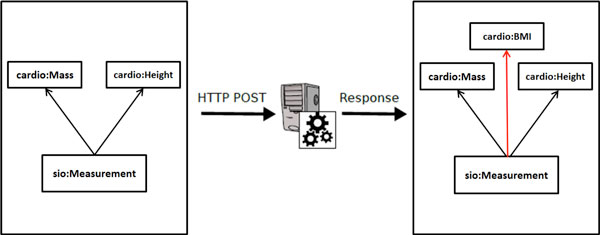
**Schematic diagram of the SADI Web Service**. SADI web service interface to the BMI calculation Service. The property-restriction imposed on the output, when detected by SHARE, triggers the discovery and invocation of the Service that attaches the BMI class with appropriate units and value properties attached to it.

It should be noted that BMI value does not exist in the data prior to running the above query and it is calculated using the Semantic Web Service schematically shown in Figure 3 (sample data, and instructions on how to send this data to the SADI service, are provided in the supplementary materials)

Once we issue the SPARQL query above, the property-restriction imposed on the output, when detected by SHARE, triggers the discovery and invocation of the Service that attaches the BMI class with appropriate units and value properties attached to it (first the service calculates all the possible output units and then it selects the one required by the output; see below for limitations of the approach). Similar our previous experiment, using this approach we were able to built ontological models that mirror the expert's classification of patients (based on BMI) of the individual clinical researcher with 100% accuracy.

### Limitations of the approach

The approach described in the examples above could reasonably be criticized as being wasteful and/or inefficient, since the unit-conversion Service converts any incoming unit into all possible output units (limited to those defined in a look-up table of the most common units used in clinical practice). This can cause performance issues; for instance a typical query takes on average 35 minutes to resolve on a single machine (4 core 3GHz machine with 8GB of memory) for the entire cohort. The problem is likely to exacerbate when dealing with large and complex and distributed datasets. Thus, the current performance of the system is not acceptable for a system designed to provide (close to) real-time support. We can envision several ways by which performance improvement can be achieved. First, we note that the apparent wastefulness explained above is not necessarily a limitation of our Semantic approach to unit representation, but rather a limitation of the SHARE client's interaction with the unit-conversion Service. The Service is capable of receiving a "desired unit" parameter during its invocation and, if present, it will use this information to configure itself to do conversions non-wastefully; converting only into that specific unit, rather than all possible units. The SHARE application, however, is not capable of passing configuration parameters to a Service during service invocation. Therefore, the apparent wastefulness of the computations is an artefact of our use of SHARE. Other SADI clients are currently under development, but were not available for this study. We reasonably anticipate that by using such clients, significant performance improvement can be achieved.

There are several other areas other areas where significant improvements could be made. This includes different strategies for SPARQL query optimizations such as parallel (as opposed to sequential) processing of different services where possible, and development of strategies to avoid invocation to irrelevant services, by checking the input and output signature (currently all the services that attach a certain property will be invoked regardless of the input and output datatype) of the services.

## Conclusions

Unit conversion is a common and troublesome barrier to integration. Busy health researchers should not need to concern themselves this trivial, error-prone, but necessary exercise. Here we have utilized a combination of semantic standards and frameworks to demonstrate, with several unit-conversion exemplar cases, that these types of data integration problems can, and should, be dealt with by the machines themselves. By encoding data with semantic transparency, it becomes possible for machines to detect unit conflicts and use semantic systems such as SADI + SHARE to automatically resolve them.

We note that a large number of measurement units in clinical practice include more complex unit patterns than the ones we modeled. For instance the majority of units used for drug dosage and clearance include temporal elements (e.g., mg/kg/hour for the drug dosage) that are not modeled in this study. To the best of our knowledge such patterns have not been modeled in any existing ontologies. As such, we plan to extend our framework to include more complex patterns together with temporal units and their conversion. Finally, we plan to extend our study to include different datasets from multiple centers and evaluate the usability of our approach in more complex biomedical scenarios.

## Additional information

Sample input/output data for SADI BMI calculator service. In order to test the BMI service using Poster Plugin, you need to have Mozilla installed on your machine. Once you installed Mozilla Firefox i go to page https://addons.mozilla.org/en-US/firefox/addon/poster/) to install the plugin. It will require that you restart the Firefox. Subsequently, go to tools->poster. In the URL tab, place the url of the service (http://cardiooroush.rhcloud.com/BMICalculator), and in the file tab browse the content of the *inputbmi.rdf *(http://cardio-soroush.rhcloud.com/inputbmi.rdf)Stored locally on your machine since the poster plugin needs the input file to be stored in a file as opposed to a url. Click on post and a pop-up window appears with the output file (should be equal to outputbmi.rdf).

## Competing interests

The authors declare that they have no competing interests.

## Authors' contributions

SS and MW planned this work, and jointly wrote this manuscript. SS executed the data migration, ontology design and extension, web service deployment, and overall analysis. BM generated and initially analyzed the source clinical dataset, and discussed and validated our approach and choice of clinical standards. All authors have read, revised and approved this manuscript.

## Declarations

Publication of this article has been funded by Special Initiatives Grant-in aid from the Heart and Stroke Foundation of British Columbia and Yukon, Microsoft Research and an Operating Grant from the Canadian Institutes of Health Research (CIHR). MDW is funded by the Isaac Peral/Marie Curie co-fund program at the Technical University of Madrid.

This article has been published as part of *BMC Medical Genomics *Volume 7 Supplement 1, 2014: Selected articles from the 3rd Translational Bioinformatics Conference (TBC/ISCB-Asia 2013). The full contents of the supplement are available online at http://www.biomedcentral.com/bmcmedgenomics/supplements/7/S1.
